# A comprehensive investigation of MoO_3_ based resistive random access memory†

**DOI:** 10.1039/d0ra03415k

**Published:** 2020-05-20

**Authors:** Jameela Fatheema, Tauseef Shahid, Mohammad Ali Mohammad, Amjad Islam, Fouzia Malik, Deji Akinwande, Syed Rizwan

**Affiliations:** Physics Characterization and Simulations Lab, Department of Physics, School of Natural Sciences (SNS), National University of Sciences and Technology (NUST) Islamabad 54000 Pakistan syedrizwan@sns.nust.edu.pk syedrizwanh83@gmail.com; CAS Key Laboratory of Magnetic Materials and Application Technology, Ningbo Institute of Materials Technology and Engineering, Chinese Academy of Sciences Ningbo 315201 China; School of Chemical and Materials Engineering, National University of Sciences & Technology (NUST) Islamabad 54000 Pakistan; College of Materials Engineering, Fujian Agriculture and Forestry University Fuzhou-350002 P. R. China; Research Centre for Modelling and Simulations, National University of Sciences & Technology (NUST) Islamabad 54000 Pakistan; Microelectronics Research Center, The University of Texas at Austin Austin Texas 78758 USA

## Abstract

The bipolar resistive switching of molybdenum oxide is deliberated while molybdenum and nickel are used as bottom and top electrodes, respectively, to present a device with resistive random access memory (RRAM) characteristics. For the trilayered structure, the SET voltage lies around 3.3 V and RESET voltage is observed to be in the −2.3 V to −2.7 V range. The conduction mechanism has been observed and revealed for the Metal–Insulator–Metal (MIM) structure which is a space-charge-limited current mechanism that follows both ohmic conduction and Child's law. Furthermore, a theoretical study has been performed by using density functional theory (DFT) to evaluate the resistance switching role of molybdenum oxide (MoO_3_). The structure has been studied with oxygen vacancy sites induced into the system which shows the reduction in bandgap, whereas an indirect bandgap of 1.9 eV and a direct bandgap of 3.1 eV are calculated for molybdenum oxide. Conclusively, the formation of a conduction filament which is fundamental for resistive switching has been explained through band structure and density of states per eV for oxygen vacancy structures of molybdenum oxide. The current work presents an in-depth understanding of the resistive switching mechanism involved in MoO_3_ based resistive random access memory devices for future data storage applications.

## Abstract 2

The bipolar resistive switching of molybdenum oxide is deliberated while molybdenum and nickel are used as bottom and top electrodes, respectively, to present a device with resistive random access memory (RRAM) characteristics.

## Graphical abstract

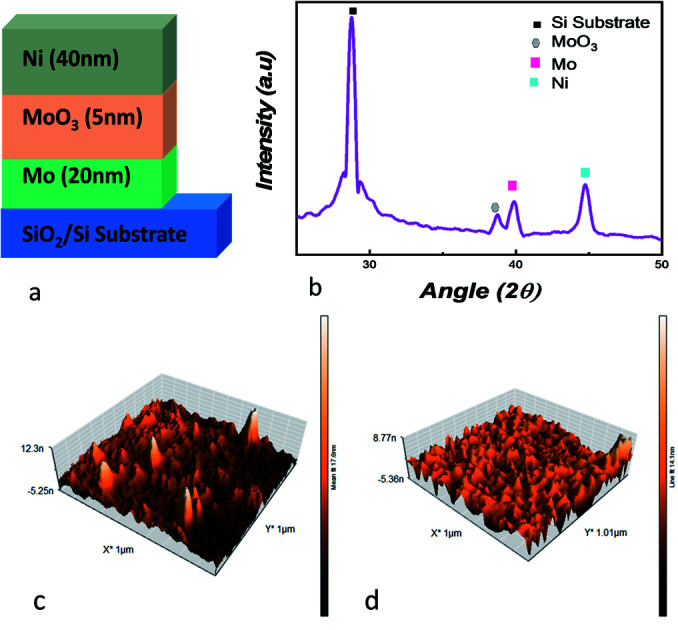

## 1 Introduction

Nowadays, non-volatile random access memory devices have gained a huge focus due to their importance in processors and are modified so that the processors can work faster with increased data storage density. There are several types of non-volatile random access memory (NV-RAM) devices *e.g.* Magnetoresistive RAM (MRAM), Ferroelectric RAM (FRAM) that have been invented in recent years.^1–3^ A lot of studies on various layered structures have been carried out with regard to their application in magnetoresistive, ferroelectric and resistive storage devices.^4–7^ During the late 20th century, resistive RAM (RRAM) was introduced as a new data storage device and in a short time, it gained a lot of attention due to its small size, high storage capacity, fast processing and superior scalability.^8–11^

Non-volatile RRAM is a combination of two metallic electrodes where an insulating layer is sandwiched between the two electrodes. Whenever a biased voltage is applied, the metal–insulator–metal (MIM) operates between two states, *i.e.* low resistance state (LRS) and high resistance state (HRS).^9,12,13^ Based on experiments, there are several types of materials proposed for middle insulating layers.^14–19^ Most of the studies are performed on binary transition metal oxides and perovskites like NiO, HfO_*x*_, TiO_2_, Al_2_O_3_ and SrTiO_3_ (ref. 20–25) as the middle layer plays a pivotal role in determining the resistive switching behavior of RRAM.

Recently, the studies on amorphous oxides for resistive switching have been reported with good results. An example of amorphous oxide is InGaZnO used as middle layer, with TiN/Ti and Pt electrodes which exhibits bipolar resistive switching with current compliance (CC) = 100 mA.^26^ Lu *et al.* has shown peculiar resistive switching characteristics of ZnSnO with higher ratio of HRS and LRS with a CC of 100 mA.^27^

The specific material used in MIM structure, its work function and band gap of insulating layer/s are important characteristics for RRAM.^12^ Takahashi *et al.* studied, the effect of electrode on Pt/Pr_0.7_Ca_0.3_MnO_3_ using different top electrodes – the electrodes used were needles' of gold, chromium, silver, platinum, tungsten and molybdenum – among which, only three electrodes were able to complete the structure for resistive switching *i.e.* chromium, molybdenum and tungsten.^28^ Yang *et al.* made a simplistic structure of MoO_*x*_ as middle layer and silver as top electrode deposited on fluorine doped tinoxide (FTO) and observed the effect of moisture on the resistive switching. The switching is observed in ±5 V for CC > 100 mA.^29^ Furthermore, it was reported that the breakdown of the device can be avoided or delayed by the use of getter layer like Ti which does not allow oxygen to escape through the system thus, increases the switching endurance of the system.^30^ This happens due to the oxygen getter tendency provided by a metallic layer which introduces oxygen defects into the insulating oxide layer.^31^ Nickel has been used as top electrode in different assortments with NiO, SiO_2_ and Al_2_O_3_ giving results of bipolar and unipolar resistive switching.^32–34^ For our device, Ni and Mo has been used as electrodes considering their high work function and resistive switching with other oxides in previous studies while using molybdenum oxide as insulating middle layer.

Moreover, a lot of experiments and theoretical analysis have been conducted, however, the phenomena of switching remains to be a motivating point of study. Resistive switching is a result of formation of conduction filament whereas, the conduction filament is formed due to oxygen vacancies in a metal oxide. The atomistic analysis of oxygen vacancies was performed for TiO_2_, NiO and HfO_*x*_/Ti, through modeling and computations to observe the outcome on conduction.^35–38^ Following a similar methodology, the study of molybdenum oxide using density functional theory is discussed in detail considering the effect of oxygen vacancy on band gap. Hence, a DFT study of molybdenum oxide is also presented here, following the experimental analysis of switching mechanism observed in Mo/MoO_3_/Ni RRAM.

## 2 Methodology

**Fig. 1:**
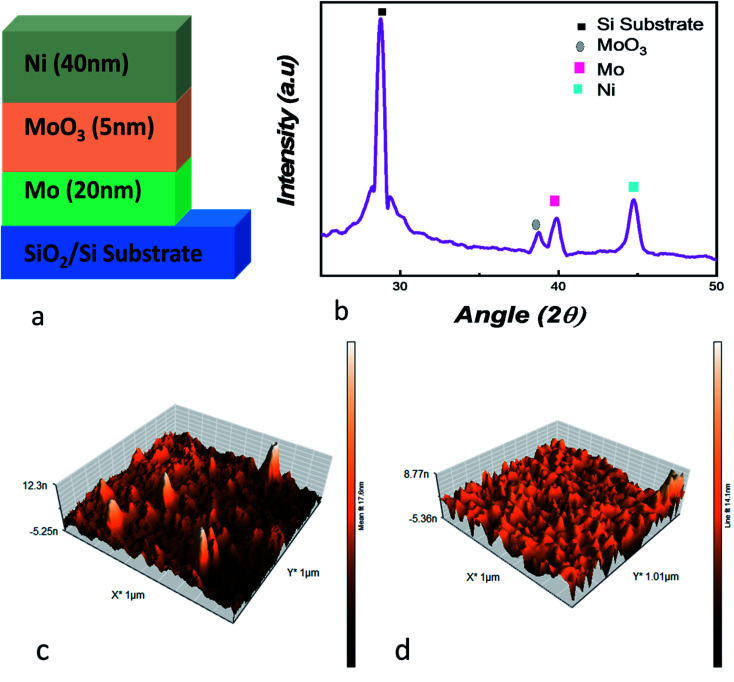
(a) Mo/MoO_3_/Ni trilayered structure deposited on SiO_2_/Si substrate, (b) XRD of the trilayered structure deposited on Si substrate, the AFM images of (c) molybdenum oxide layer (d) Ni layer are also shown.

For the deposition of Mo/MoO_3_/Ni on silicon substrate (Fig. 1a), the first step was deposition of 20 nm Mo layer, deposited through direct current (DC) sputtering. The base pressure and working pressure of the vacuum chamber was 7.0 × 10^−4^ Pa and 1.0 × 10^−1^ Pa. Highly pure target of Mo was used, and for the purpose of uniform deposition the substrate was kept on rotation. Ar flux was maintained at 60 sccm through the deposition process. For deposition of 5 nm thin layer of molybdenum oxide the reactive sputtering technique was used in Ar–O_2_ plasma at 60 sccm and 20 sccm flow rate respectively with 120 W. The Mo/MoO_3_ deposition was *in situ* while Ni was deposited *ex situ* using electron beam physical vapor deposition technique. The substrates with the deposited layers of molybdenum and molybdenum oxide were loaded into the vacuum chamber where the chamber was evacuated to a base pressure of 1.6 × 10^−4^ Pa. During the deposition, the voltage, deposition rate, pressure and current were 6 kV, 1 Å s^−1^, 4 × 10^−4^ Pa and 120 mA respectively to form a 40 nm layer of Ni.

For analysis of the Mo/MoO_3_/Ni trilayered structure, X-ray diffraction (XRD) and atomic force microscopy (AFM) were used. The Bruker D8 Advance and Nanosurf Naio AFM systems were used for XRD AFM, respectively. The current–voltage characteristics were obtained using Keysight B2900A SMU precision system with high-voltage point was connected to the top Ni electrode while the ground point was connected to the bottom electrode, *i.e.* Mo and substrate interface.

For the exploration of molybdenum oxide structure at atomic level, a density functional theory based study was conducted through WIEN2k employing Kohn–Sham equations while the chosen basis set is full-potential linear augmented plane wave method.^39,40^ Structure of molybdenum oxide MoO_3_ was optimized and minimized at 300 *k*-points in the Irreducible Brillouin Zone (IBZ) which corresponds to a *k*-mesh of 2 × 10 × 10. Generalized Gradient Approximation (GGA) with Perdew–Burke–Ernzerhof (PBE) was employed as exchange correlation functional for geometric and electronic parameter determination.^41–43^

## 3 Results and discussions

### 3.1 Experimental analysis

For physical assessment, XRD of the trilayered structure is shown in Fig. 1b, where the diffraction peaks confirm the presence of the layers that were deposited on to the substrate. For angles 2*Θ* = 28.76°, 38.61°, 39.7° and 44.76°, four peaks are observed, representing substrate peak, MoO_3_, Mo and Ni, respectively.^44–46^ At 38.61°, the plane detected for molybdenum oxide is (600) with JCPDS card #03-065-2421.^47,48^ This shows us the crystallographic nature of the device. For the surface topography, Fig. 1c and d shows the AFM image, where the root mean square roughness was calculated to be 1.336 nm after the deposition of molybdenum oxide layer over Mo. The roughness is suitable considering the deposition of 5 nm thin layer deposited through reactive dc-sputtering technique. Whereas, Fig. 1d shows the AFM image for the Ni layer deposited on the oxide layer with a root mean square roughness of 1 nm.

The resistive random access memory consists of a trilayered structure where the middle layer is an insulating oxide, and, top and bottom layers are metallic electrodes. The phenomena of the conduction has been explained by the formation of oxygen ions and oxygen vacancies as a consequence of rupturing in the middle layer to form a conduction filament (CF) as depicted in Fig. S1.†^31^ After the formation of trilayered structure, a biased voltage is applied to the top electrode while the bottom electrode is grounded. With the increase of voltage, oxygen ions migrate from middle layer to the top electrode leaving behind a filament of oxygen deficient sites called as conduction filament (CF).^12,31^ At the voltage point where filament is completely formed, a swift rise in current occurs and reaches the current compliance limit; the voltage at this point is called “SET” voltage. Here, the device is switched from a HRS to LRS and stays in LRS until “RESET” voltage is achieved where the CF consisting of oxygen deficient sites are filled up by oxygen ions which were travelled back from the top electrode. This is the result of negative voltage and the device is switched from LRS to HRS. Since the device is switched to HRS at RESET voltage, it retains the state until a SET voltage is applied where it goes back to the LRS and the cycle goes on between SET and RESET voltage. The switching behavior where the SET and RESET voltage lies in separate quadrants is the bipolar resistive switching as shown in Fig. S1.†

**Fig. 2:**
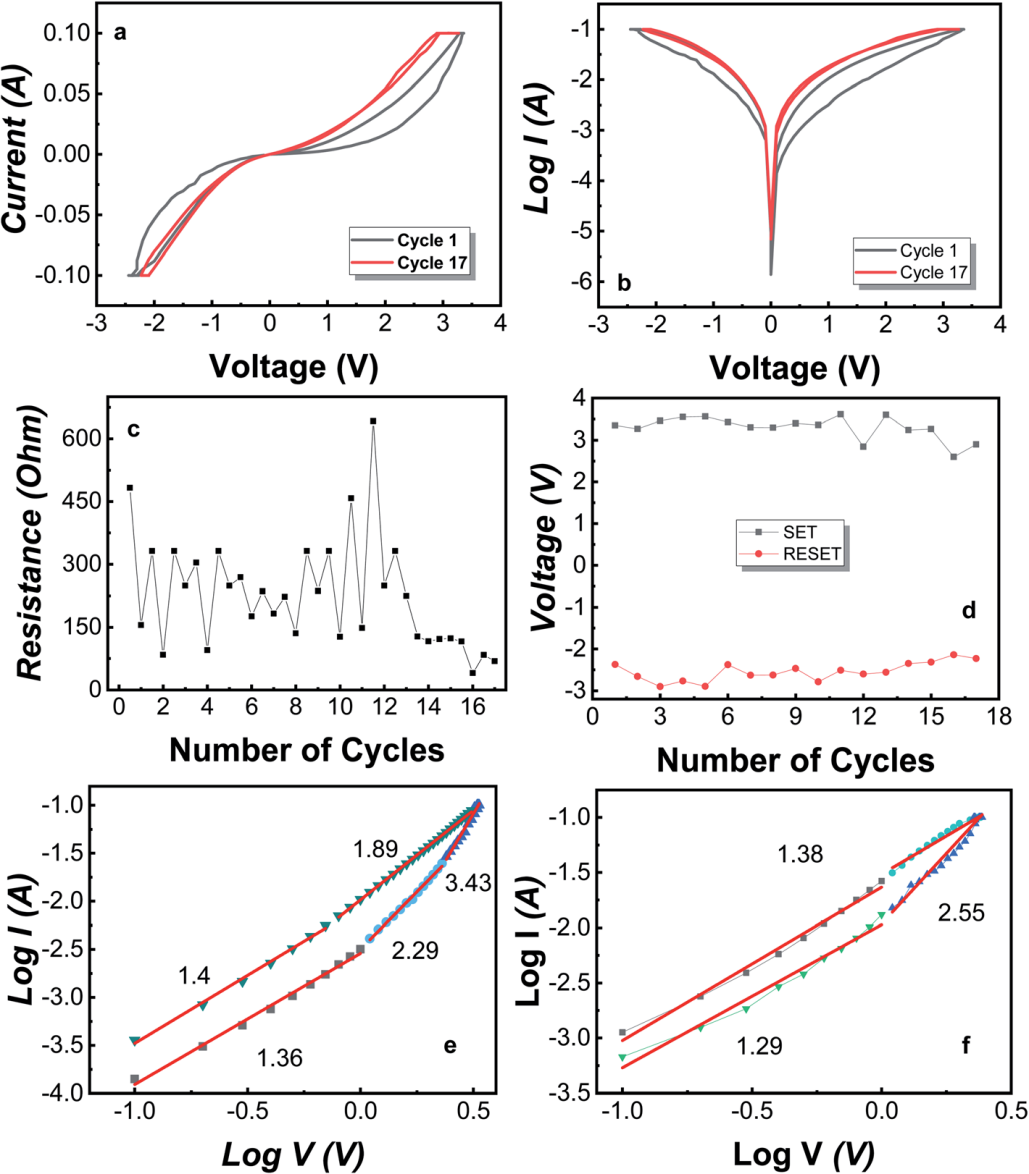
Bipolar switching characteristics of Mo/MoO_3_/Ni showing resistive switching in cycle 1 and 17 through (a) current–voltage (*I*–*V*) (b) logarithmic current–voltage (log *I*–*V*) graph (c) retention characteristics of Mo/MoO_3_/Ni (d) SET and RESET voltages plotted against the number of cycles. Double logarithmic graph of first (e) positive and (f) negative cycle with linear fitting and slope values indicating SCLC mechanism.

For the MIM Mo/MoO_3_/Ni structure, the bipolar resistive switching for the first and last cycle is shown in Fig. 2a and b, where the applied voltage cycle goes from 0 V → +3.3 V → 0 V → −2.7 V → 0 under the current compliance (CC) is kept at 100 mA. Recently, certain devices have reported to show an electroforming-free process.^50–54^ The forming voltage was not required for our device, *i.e.* there was no electroforming process needed. Electroforming process needs an initial high voltage compared to SET/RESET voltage and is a destructive chemical process.^49^ As the biased voltage is applied to the top electrode (Ni), the oxygen from molybdenum oxide converts to O^2−^ oxygen ions and vacancy sites, where oxygen ions move towards the top electrode as the voltage applied is positive and leaves behind a CF of oxygen vacancies.

Yang *et al.* have discussed the formation of silver oxide at silver/MoO_3_ interface of the device fabricated on FTO substrate.^55^ The study of bilayer RRAM consisting of MgO/MoO_3_ in the middle layer with Mg electrodes shows that during the switching, magnesium layer becomes oxidized as the oxygen ions move towards the electrode.^56^ Tan *et al.* have fabricated Au/MoO_3_/Au on Si substrate and have discussed that during the switching from HRS to LRS, MoO_3_ is converted to MoO_2_ which is conductive and MoO_2_ becomes MoO_3_ when switching from LRS to HRS.^57^ For our device, the biased voltage results in the oxidization of Ni at the Ni/MoO_3_ interface as well as formation of MoO_2_ from where, oxygen leaves behind the Mo ions. This results in a conductive middle layer. The observed band gap for MoO_3_ is 3.2 eV while MoO_2_ is reported to be a conductor.^58,59^ Also, the conversion between MoO_3_ and MoO_2_ has been reported.^60–62^ At SET voltage, the CF is formed completely and the device switches from HRS to LRS. For cycle 1, the switching happens at ∼3.3 V. Once the CF is formed, the device stays in LRS unless it reaches the RESET voltage, *i.e.* −2.4 V and the device shifts into HRS. During this switching, oxygen migrates from the Ni/MoO_3_ interface back towards the Mo atoms and the CF is filled-up moderately by the formation of MoO_3_. The filament starts to fill up resulting in soft breakdown of the CF which changes the device from LRS to HRS. The device remains in HRS or LRS unless a set or reset voltage is applied, respectively.

Fig. 2c illustrates the HRS and LRS throughout the cycles *i.e.* 1 to 17, where the read voltage is 0.5 V such that first point corresponds to the HRS and second point corresponds to LRS of each cycle. As this device is demonstrating bipolar switching, the reset voltage is negative and lies in the range of −2.3 V to −2.9 V for 16 cycles. Fig. 2d shows the SET and RESET voltage for each cycle of the device. Furthermore, at any point, by applying a small voltage of ∼0.5 V, the state observed state will be HRS or LRS. For the Mo/MoO_3_/Ni structure, the breakdown of the CF happens after cycle number 16 until which, we observe the bipolar resistive switching. At the breakdown point, the CF remains intact and the MIM structures resists the shift to HRS, hence staying in the LRS. The breakdown of the device occurs due to loss of oxygen ions from the system, which can be controlled by the addition of a getter layer. The getter layer is of such a compound that prevents the oxygen from leaving the system.

For the conduction of oxygen ions, a specific mechanism takes place which is identified by the slope values obtained from linear fitting of double logarithmic graphs shown in Fig. 2e and f. Space-charge-limited current (SCLC) mechanism is observed in several RRAM structures which consists of three major conduction mechanisms namely, (i) ohmic conduction (ii) Child's law and (iii) steep rise in current region.^63–65^ The equation for SCLC is

1 *I* = *αV* + *βV*^2^

For ohmic conduction, the slope values are ∼1, for conduction through Child's law, the slope value approximates to 2 and is greater in the region where there is a steep rise in current which describes three possible regions in SCLC mechanism. A double logarithmic plot of logarithmic current against logarithmic voltage is plotted in Fig. 2e and f corresponding to the positive voltage and negative voltage of the first cycle. The slopes obtained from Fig. 2e and f after linear fitting of the double logarithmic plots. It is observed that SCLC mechanism is present throughout the cycle. From Fig. 2e, the slope values are defined over 4 regions. The slope = 1.36 identifies ohmic conduction, 2.29 complements the Child's law, and 3.43 is the region with steep rise in current corresponding to SCLC mechanism. For LRS, the slope values are 1.8 and 1.4 indicating verification of Child's law and ohmic conduction.^63^ For Fig. 2f, the slope values are 1.29, 2.55 for HRS, representing ohmic conduction and Child's law respectively and then is 1.38 for LRS of negative cycle which corresponds to the ohmic conduction. Overall, the conduction mechanism observed is SCLC and for further understanding of the CF, DFT analysis is performed for MoO_3_ unit cell and its supercell with induced oxygen vacancies.

### 3.2 Computational analysis

**Fig. 3:**
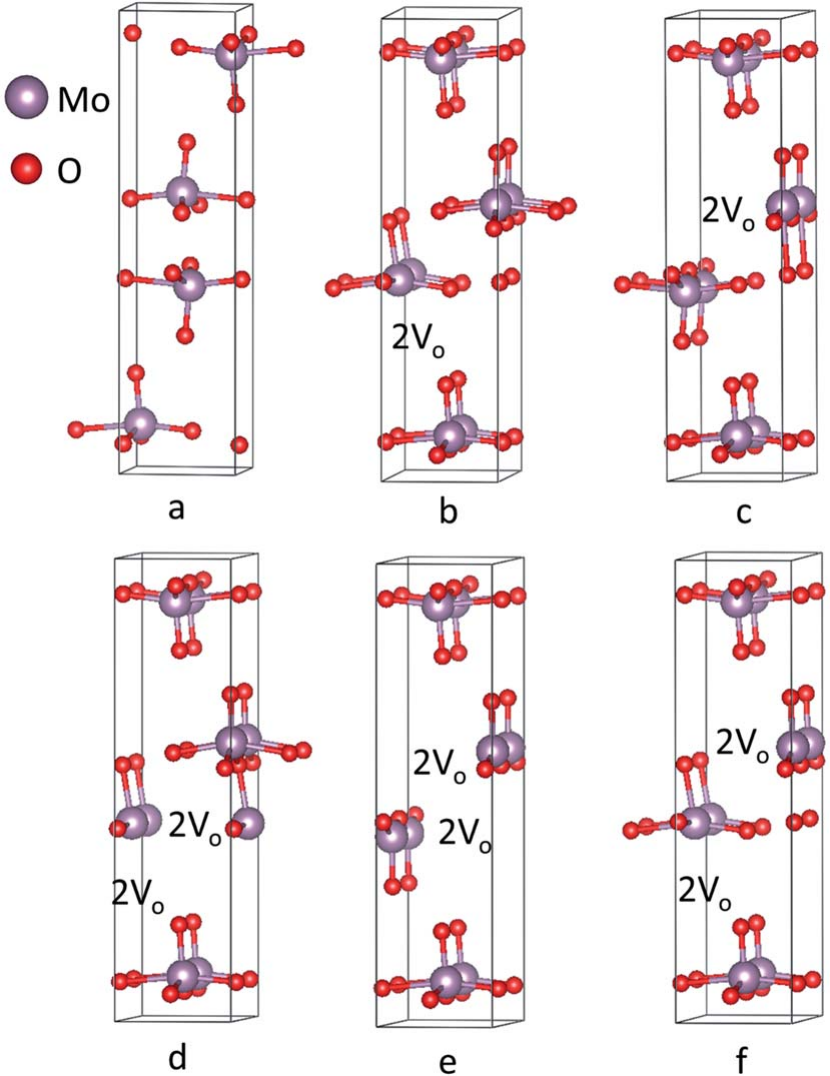
(a) Crystal structure of molybdenum oxide (MoO_3_) calculated at LDA-GGA level of theory. Structure of molybdenum oxide with vacancies along (100) (b) vacancy 1 (c) vacancy 2 (d) vacancy 3 (e) vacancy 4 (f) vacancy 5.

Crystal geometry of molybdenum oxide studied is orthorhombic, with space group #62 *Pnma*. Based on respective space group, lattice parameters of MoO_3_ has values of *a* = 14.845 Å, *b* = 3.705 Å, and *c* = 3.908 Å with *α* = *β* = *γ* = 90° depicted in Fig. 3a obtained after the relaxation of lattice parameters which are in close agreement with previous results.^66^ After relaxation of the atomic positions of MoO_3_, unit cell consists of one Mo atom and 3 inequivalent oxygen atoms with the following atomic co-ordinates: Mo (0.09610287, 0.25, 0.06968197), O1 = (0.06120865, 0.75, 0.99865431), O2 = (0.08179154, 0.25, 0.51727228) and O3 = (0.20449652, 0.25, 0.03048734).

Recently, Köpe *et al.* had demonstrated a study of titanium oxide with oxygen vacant sites in different alignment and arrangement which showed that the oxygen vacant sites are aligned in a certain order that can be the possible ON states of titanium oxide based RRAM.^38^ This work, demonstrates crystal structures with varying oxygen vacant sites in a supercell. Computationally, a supercell of 1 × 2 × 1 was generated for studying the effect of oxygen deficiency defect by introducing oxygen vacancy sites in the plane (100) depicted in Fig. 3b–f corresponding to vacancy 1–5 structures, respectively. For vacancy 1 and 2, there are 2 consecutive oxygen vacancies along the same plane. For vacancies 3 and 4, 2 oxygens are removed from single molybdenum leading to production of 4 adjacent oxygen vacancy sites simulating a conduction filament. Vacancy 5 structure has also shown 4 oxygen vacancy sites but they are not adjacent. These supercell structures were minimized using 60 *k*-points with 1 × 4 × 7 *k*-mesh in IBZ. Furthermore, the SCF, bandstructure and DOS for the unit cell of MoO_3_ was performed with 300 *k*-points whereas for oxygen vacancy structures of MoO_3_, the number of *k*-points used were 100.

**Fig. 4:**
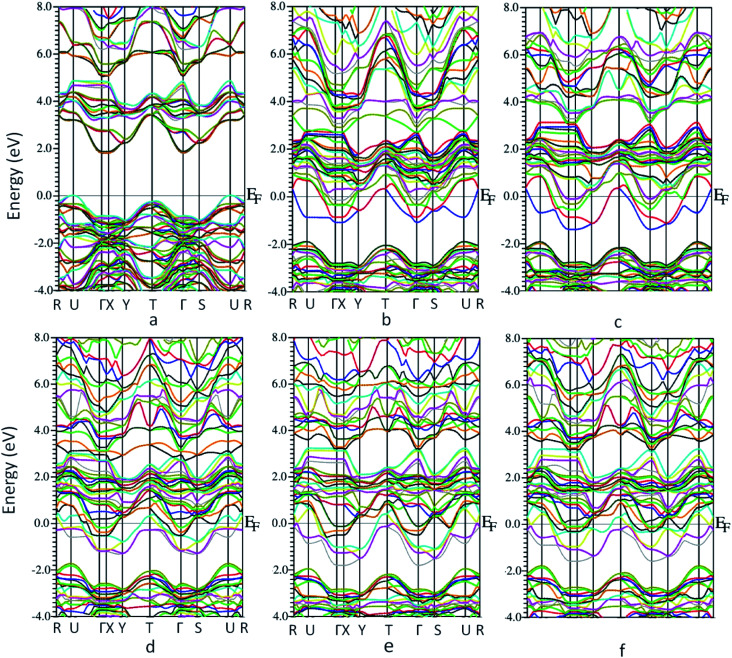
Bandstructure of molybdenum oxide in 1 × 2 × 1 supercell (a) no oxygen vacancy (b) vacancy 1 (c) vacancy 2 (d) vacancy 3 (e) vacancy 4 (f) vacancy 5.

The bandstructure for MoO_3_ supercell without any vacancy sites and for the 5 vacancy structures is shown in Fig. 4a–f. Fig. 4a, shows that there is an indirect band gap of 1.9 eV from U to Γ point, whereas a direct band gap of 3.1 eV at U-point in the Brillouin zone. As the oxygen vacancy is introduced, the behavior of the bandstructure shifts towards metallic from insulator. We observe an overlap of bands around the Fermi level (Fig. 4b–f) as the bandgap reduces to zero.

**Fig. 5:**
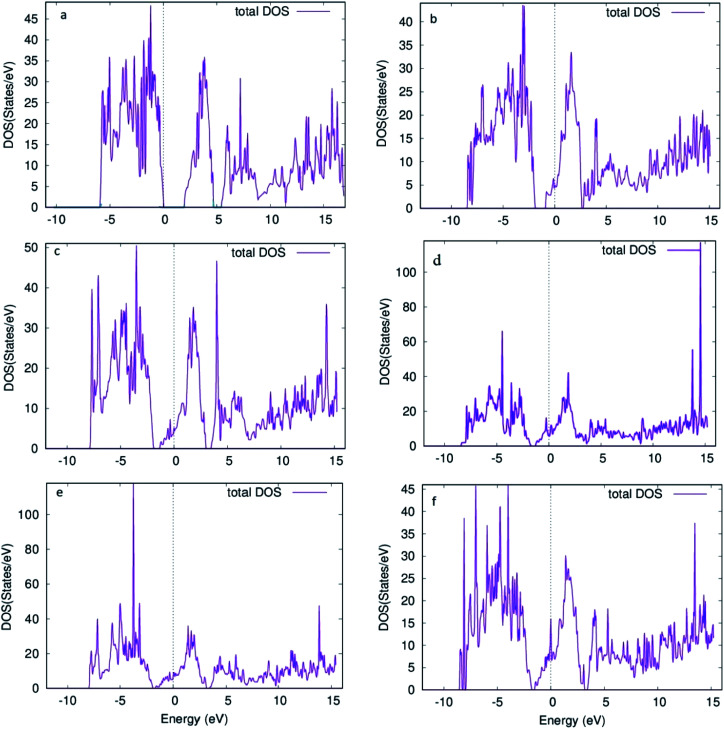
Density of states for supercell 1 × 2 × 1 structure of molybdenum oxide (MoO_3_) (a) no vacancy (b) vacancy 1 (c) vacancy 2 (d) vacancy 3 (e) vacancy 4 (f) vacancy 5.

Furthermore, the total density of states (DOS) for the 6 structures shows us the number of states per eV in Fig. 5. For Fig. 5a, a small gap is observed corresponding to the band gap of the structure. Introducing oxygen vacancy sites leads to a small shift that takes place for the states in the conduction band to upper edge of valence band (Fig. 5b and c). Moreover, total DOS for structures with 4 adjacent oxygen vacancy sites, *i.e.*Fig. 5d and e, revealed high peaks in both conduction and valence bands along with a good number of states present at Fermi level. Hence, introduction of oxygen vacancy sites resulted in metal-like behavior of molybdenum oxide in RRAM. Likewise, DOS calculated for vacancy 5 structure (Fig. 5f), the DOS behavior is almost similar to the DOS of vacancy 1 and vacancy 2 structures (Fig. 5b and c). For the individual contributions of the atoms to the total DOS, the density of states for Mo and O atoms of the % vacancy based structures is observed and the situation is clarified further. In Fig. S2,† we see that the top of the valence band is occupied by O and bottom of the conduction band is occupied by Mo for the unit cell of MoO_3_. From Fig. S3, S5, S7, S9 and S11,† it is clearly observed that for the Mo whose bond with oxygen was broken and readjusted, the DOS per eV has changed and shifted towards the Fermi level, in the supercell with oxygen vacancy sites as well as increase in intensity of certain peaks of DOS for Mo in Fig. S4.† Similarly, DOS of oxygen for the vacancy structures, shown in Fig. S4, S6, S8, S10 and S12,† shows the shifting of the peaks for O that is attached to the Mo surrounding the oxygen deficient sites.

At SET voltage, as the device switches from HRS to LRS, conductive MoO_2_ is formed due to migration of oxygen ions and at RESET voltage, switching from LRS to HRS, MoO_2_ converts to MoO_3_.^57^ From the computational analysis of MoO_3_, we can say that under an applied electric field or biased voltage, the oxygen ions move towards the top electrode, leaving behind vacant sites in molybdenum oxide which reduces the band gap of the middle layer and the layer becomes conductive. Hence, it is observed that oxygen vacancies play a vital role in the formation of conduction filament and are necessary for the trilayered structure to achieve a low resistance state.

## 4 Conclusion

In summary, bipolar resistive switching is observed in Mo (20 nm)/MoO_3_ (5 nm)/Ni (40 nm) structure deposited on silicon substrates, where the entire thickness of the trilayered structure is observed to be 65 nm. The AFM and XRD study was done to analyze surface morphology and crystallographic nature of the deposited layers. The retention of the MIM structure was observed to last for 17 cycles in the voltage range of 3.3 V to −2.8 V and CC = 100 mA. The endurance of the Mo/MoO_3_/Ni can be increased by introduction of a getter layer. The conduction mechanism was analysed which shows the clear presence of SCLC, where oxygen ions are source of conduction in the MIM structure. The structural analysis of MoO_3_, through oxygen vacancy defective structures in supercell, using DFT, showed that the oxygen vacancies change the band gap such that the insulating molybdenum oxide becomes metallic. And ultimately, through the DOS analysis, it was concluded that the oxygen vacancy sites have an effect on the DOS of surrounding atoms resulting in the reduction of bandgap. Hence, it is observed that the oxygen vacancy sites resulting in conduction filament are the reason for HRS and LRS in molybdenum oxide.

## Conflicts of interest

There are no conflicts of interest to declare.

## Supplementary Material

RA-010-D0RA03415K-s001
