# Supplementary material for: A comprehensive investigation of MoO3 based resistive random access memory
Source: RSC Adv. 2020 May 20;10(33):19337–45. doi: 10.1039/d0ra03415k (PMC9054044; doi:10.1039/d0ra03415k)
Supplement: RA-010-D0RA03415K-s001 [file RA-010-D0RA03415K-s001.pdf]

Electronic Supplementary Material (ESI) for RSC Advances.
This journal is © The Royal Society of Chemistry 2020

A Comprehensive Investigation of MoO_3 based Resistive Random Access Memory

Supplementary Information

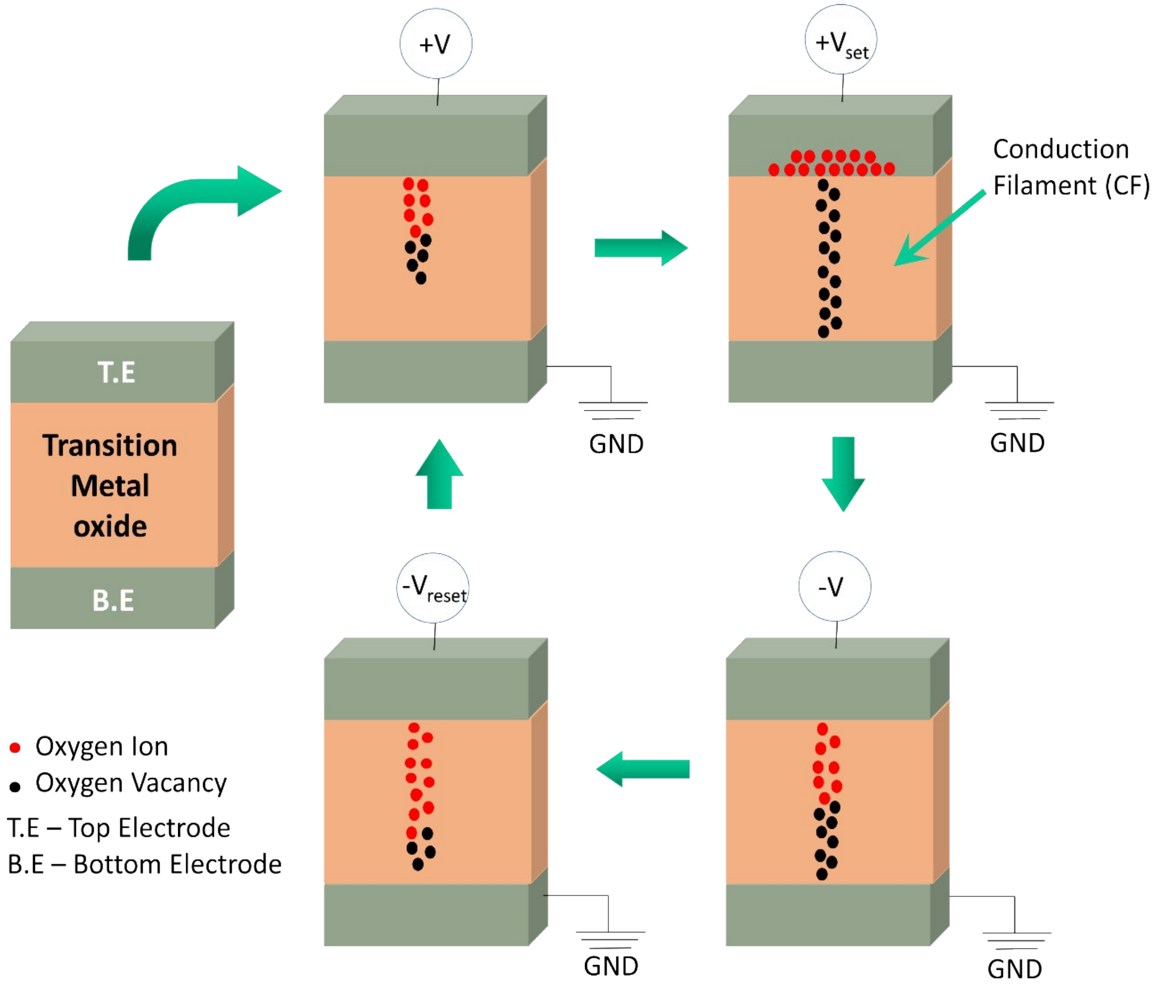

Figure S1 Schematic illustration of bipolar switching mechanism in MIM structure behaving as RRAM

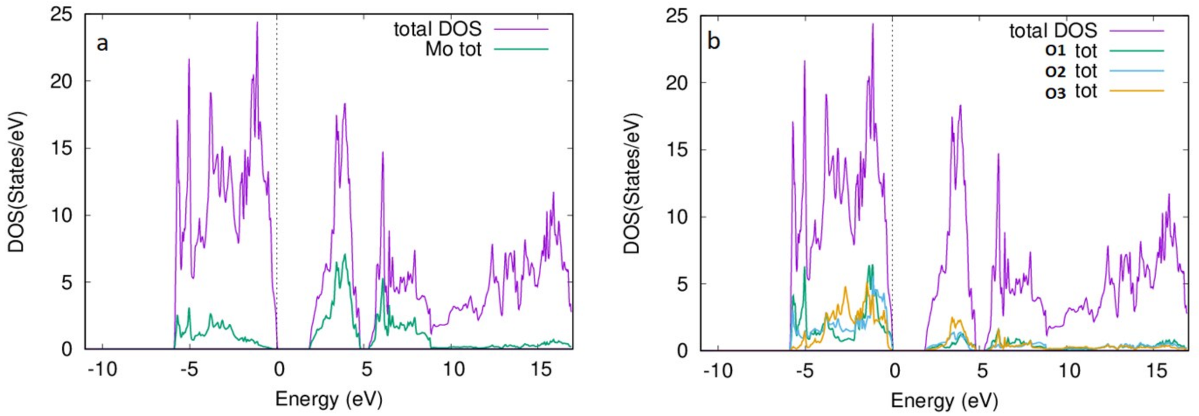

Figure S2 Density of states for molybdenum oxide (MoO_3) in unit cell

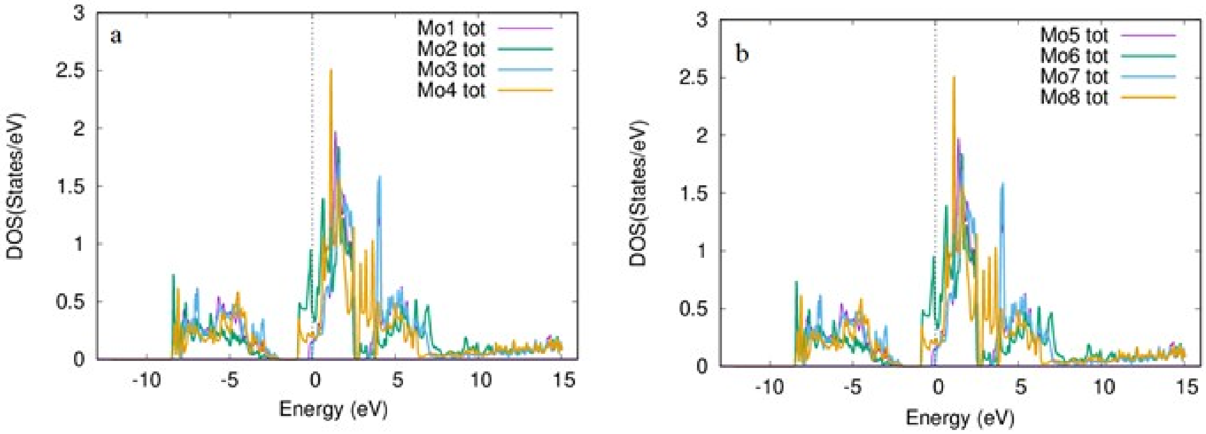

Figure S3 Total Density of states for Mo in Vacancy 1 structure of molybdenum oxide (MoO_3) supercell

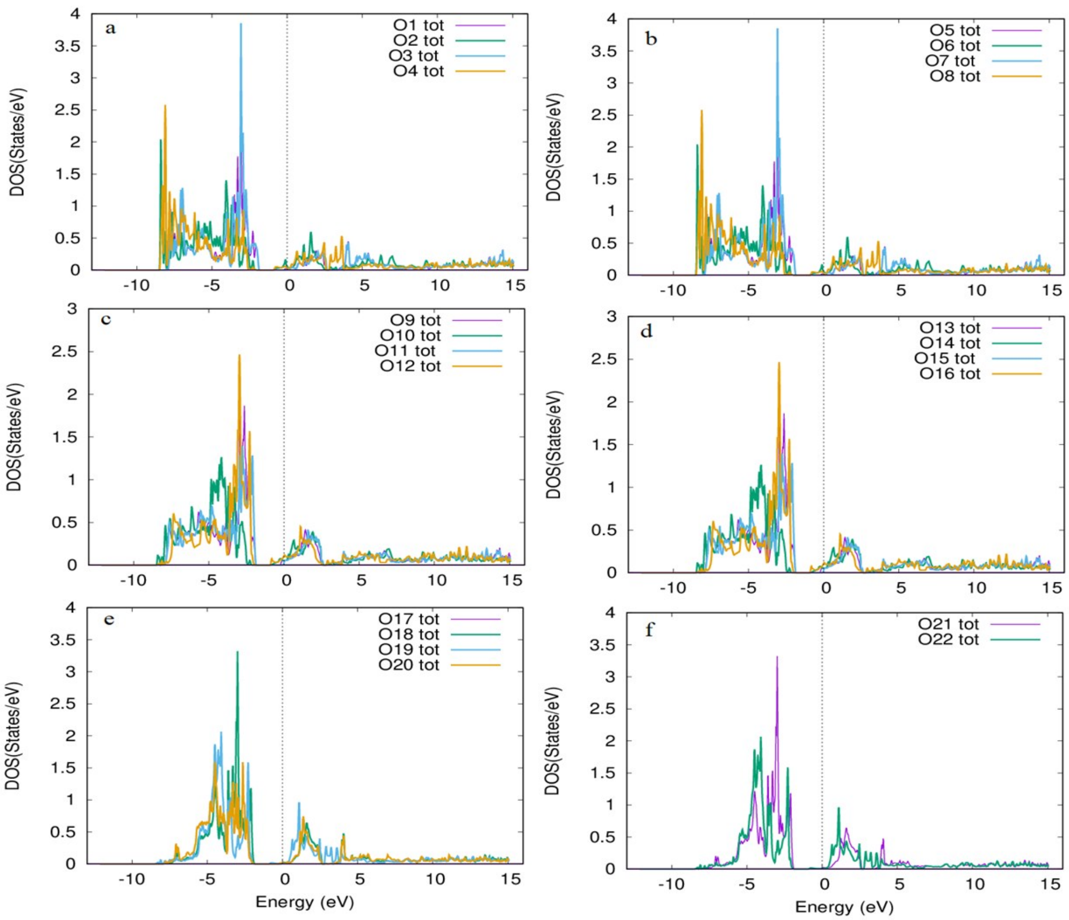

Figure S4 Total Density of states for O in Vacancy 1 structure of molybdenum oxide (MoO_3) supercell

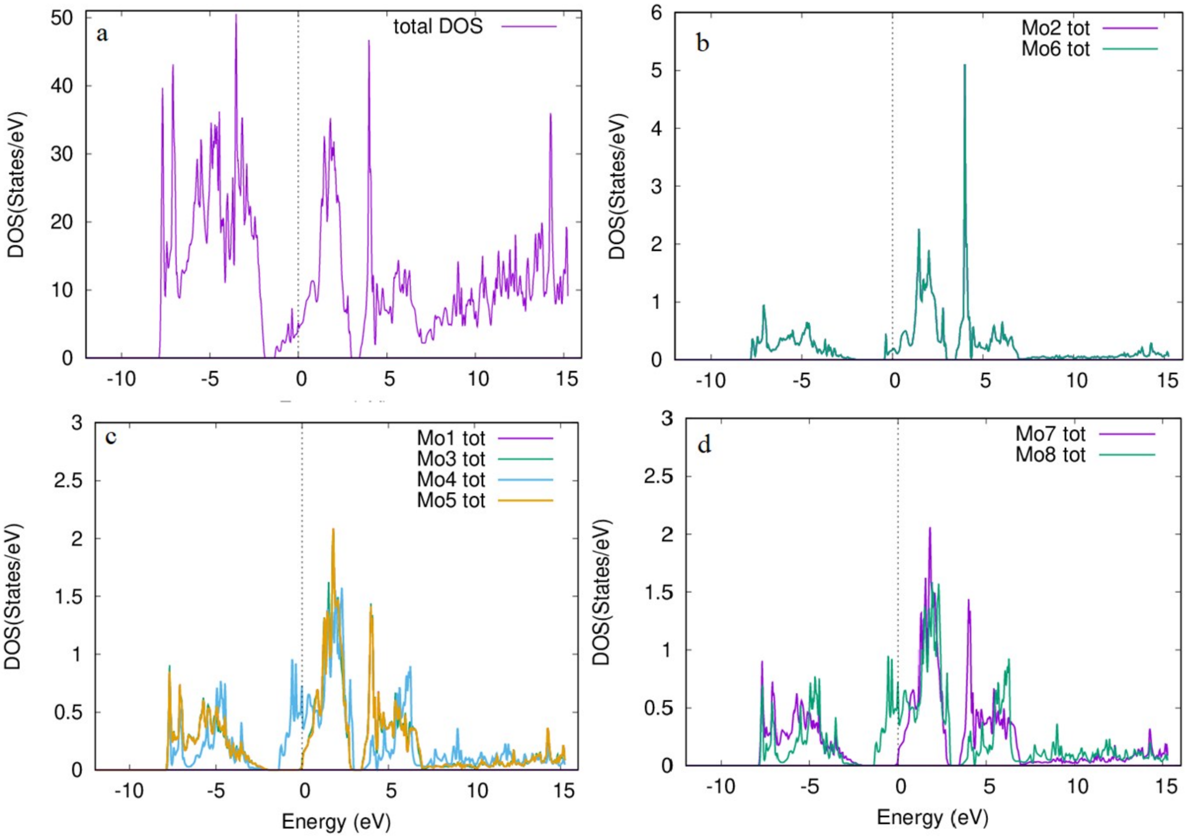

Figure S5 Total Density of states for Mo in Vacancy 2 structure of molybdenum oxide (MoO_3) supercell

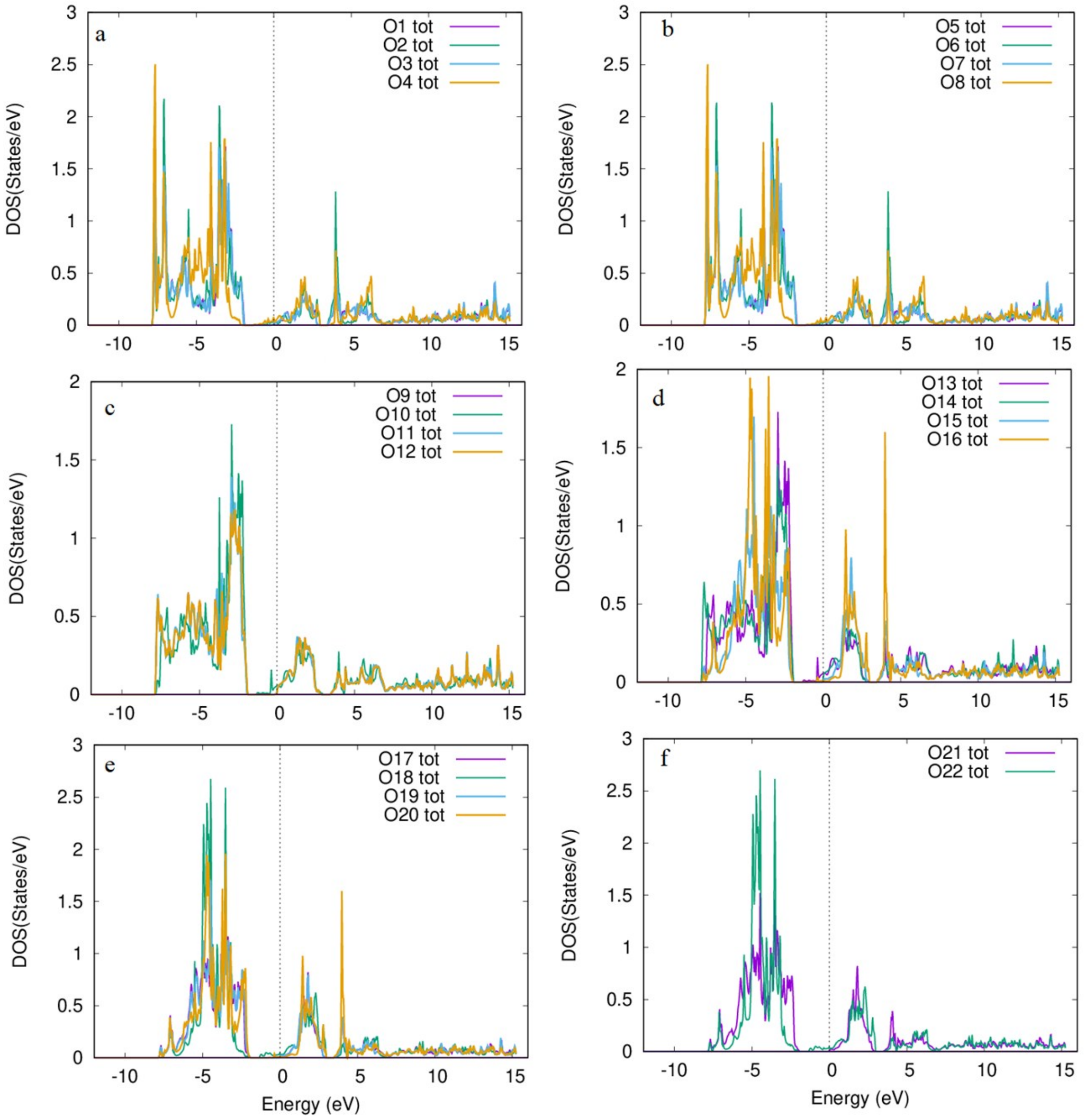

Figure S6 Total Density of states for O in Vacancy 2 structure of molybdenum oxide (MoO_3) supercell

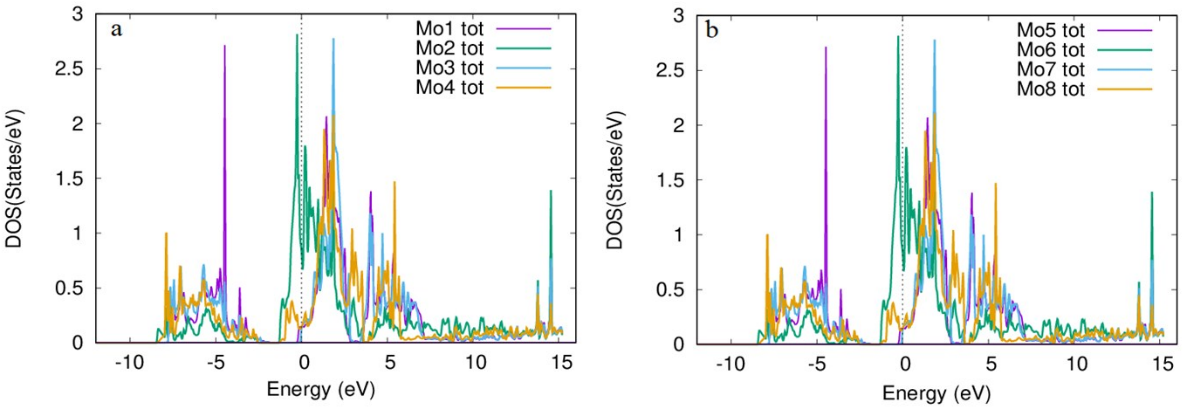

Figure S7 Total Density of states for Mo in Vacancy 3 structure of molybdenum oxide (MoO_3) supercell

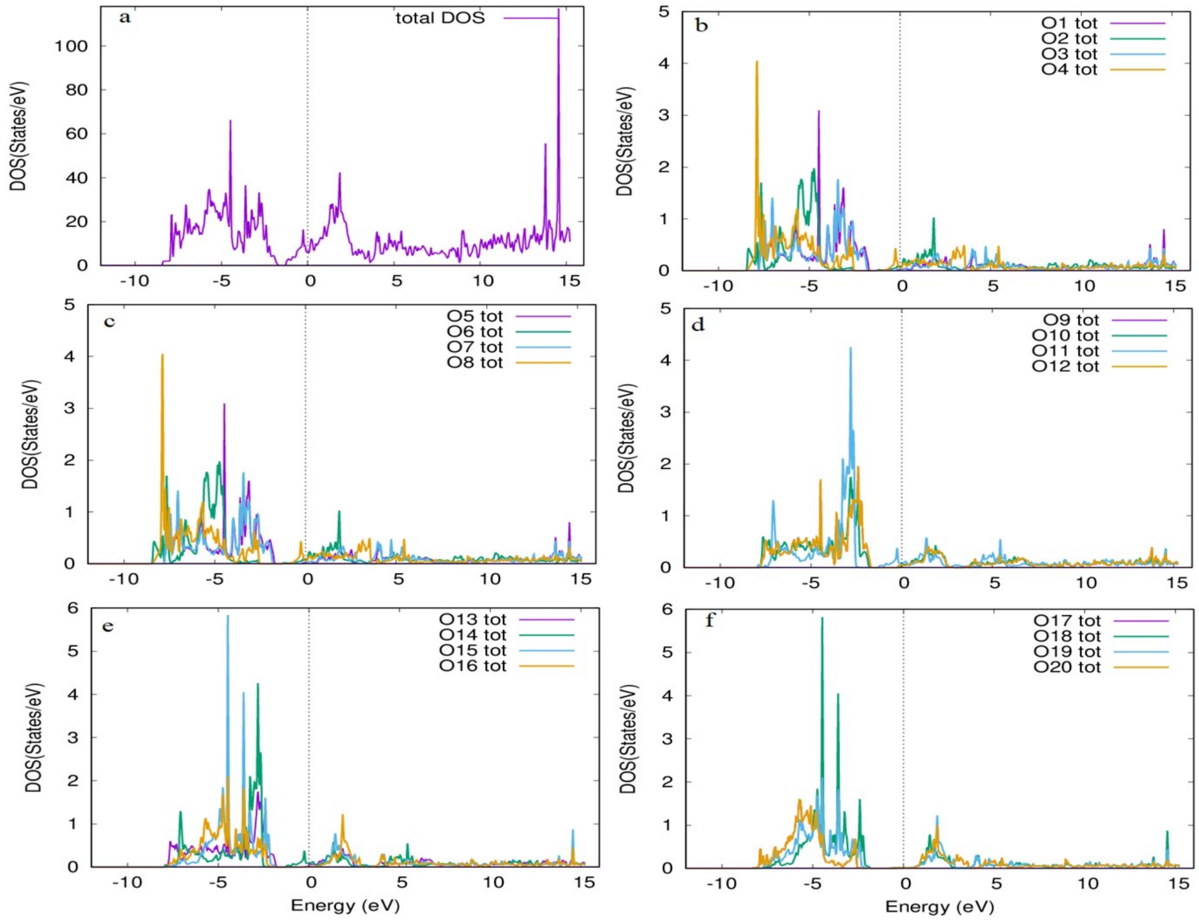

Figure S8 Total Density of states for O in Vacancy 3 structure of molybdenum oxide (MoO_3) supercell

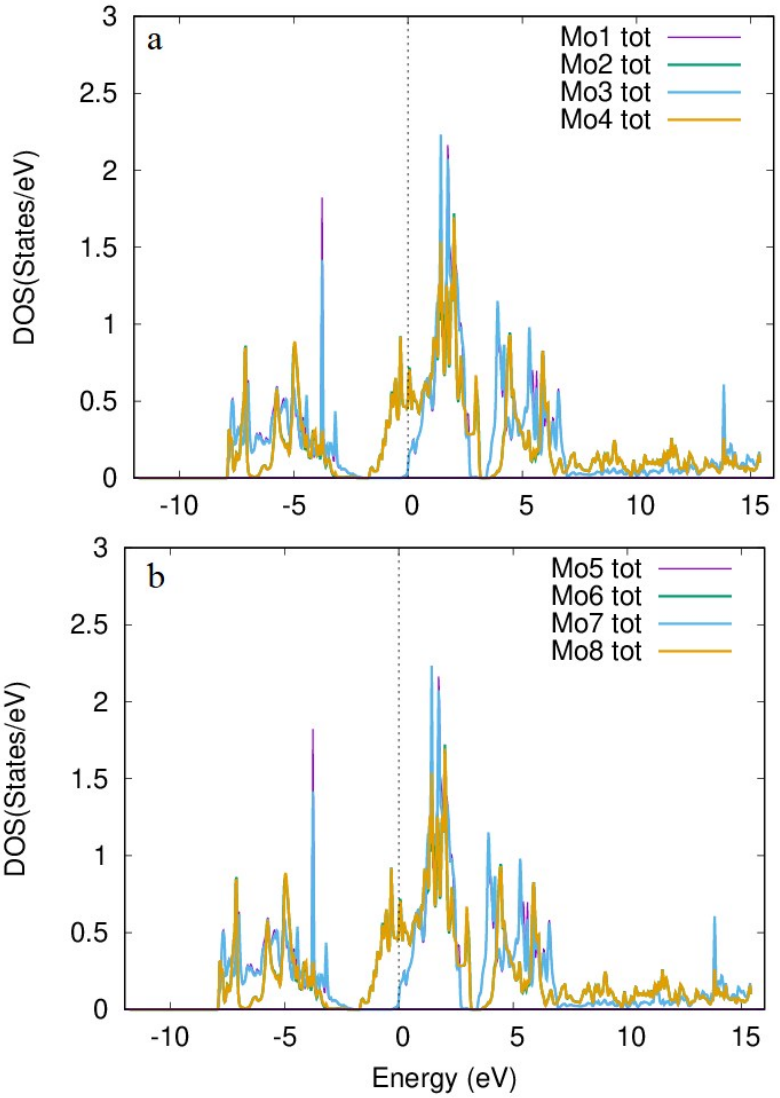

Figure S9 Total Density of states for Mo in Vacancy 14 structure of molybdenum oxide (MoO_3) supercell

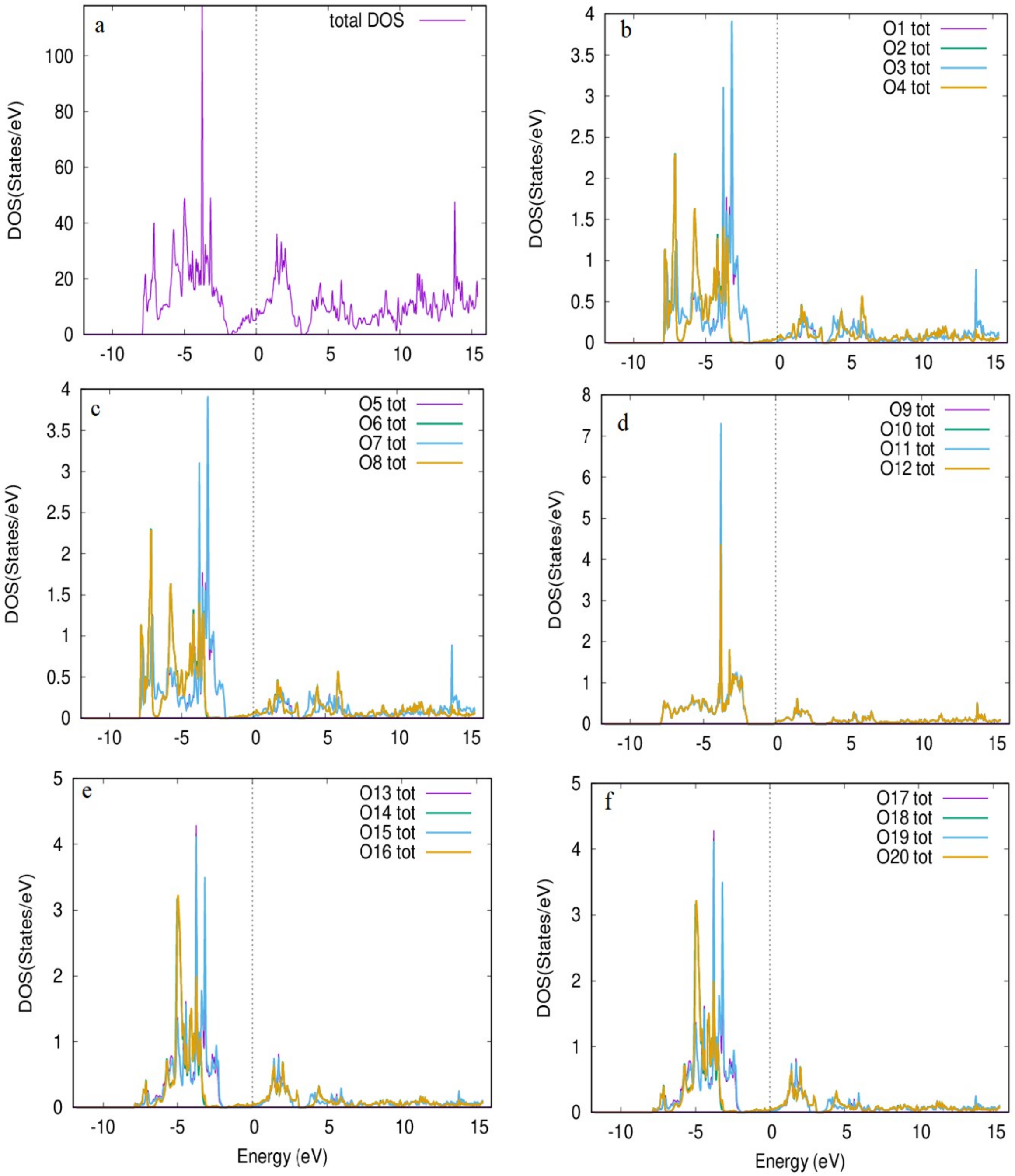

Figure S10 Total Density of states for O in Vacancy 4 structure of molybdenum oxide (MoO_3) supercell

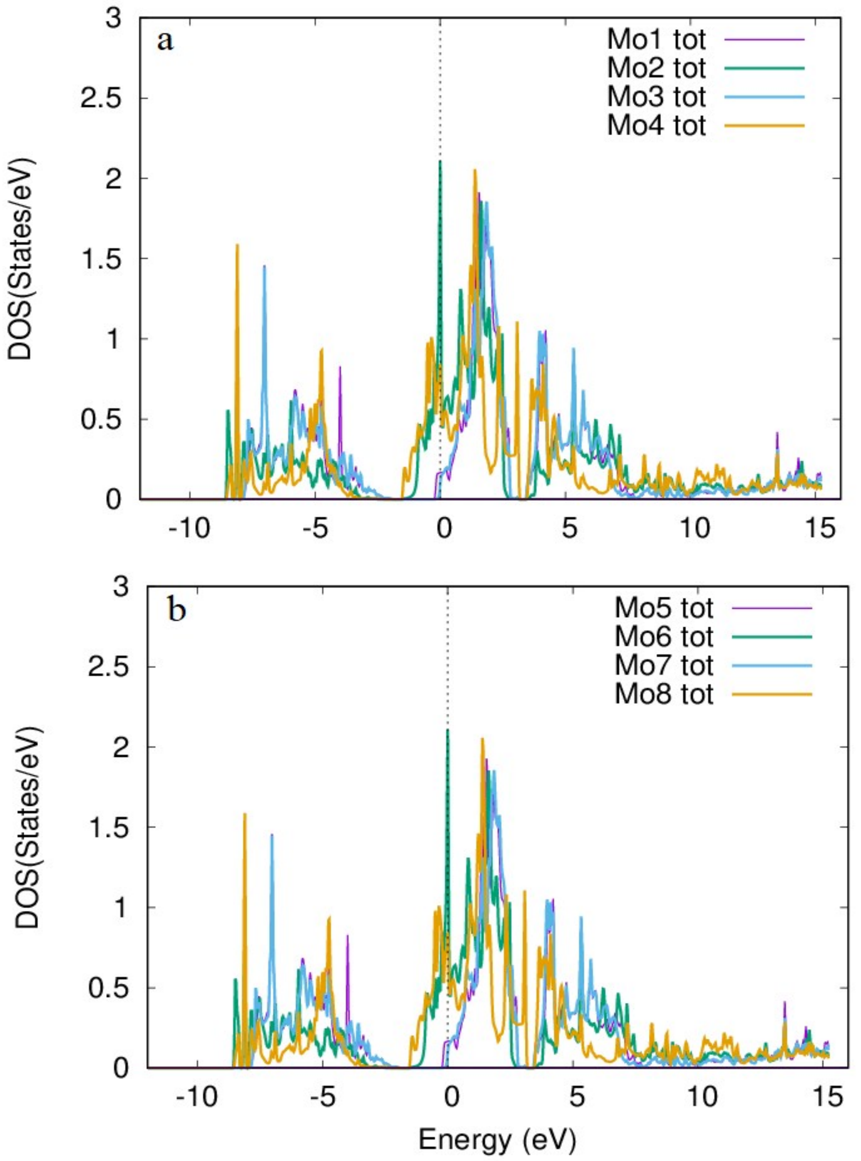

Figure S11 Total Density of states for Mo in Vacancy 5 structure of molybdenum oxide (MoO_3) supercell

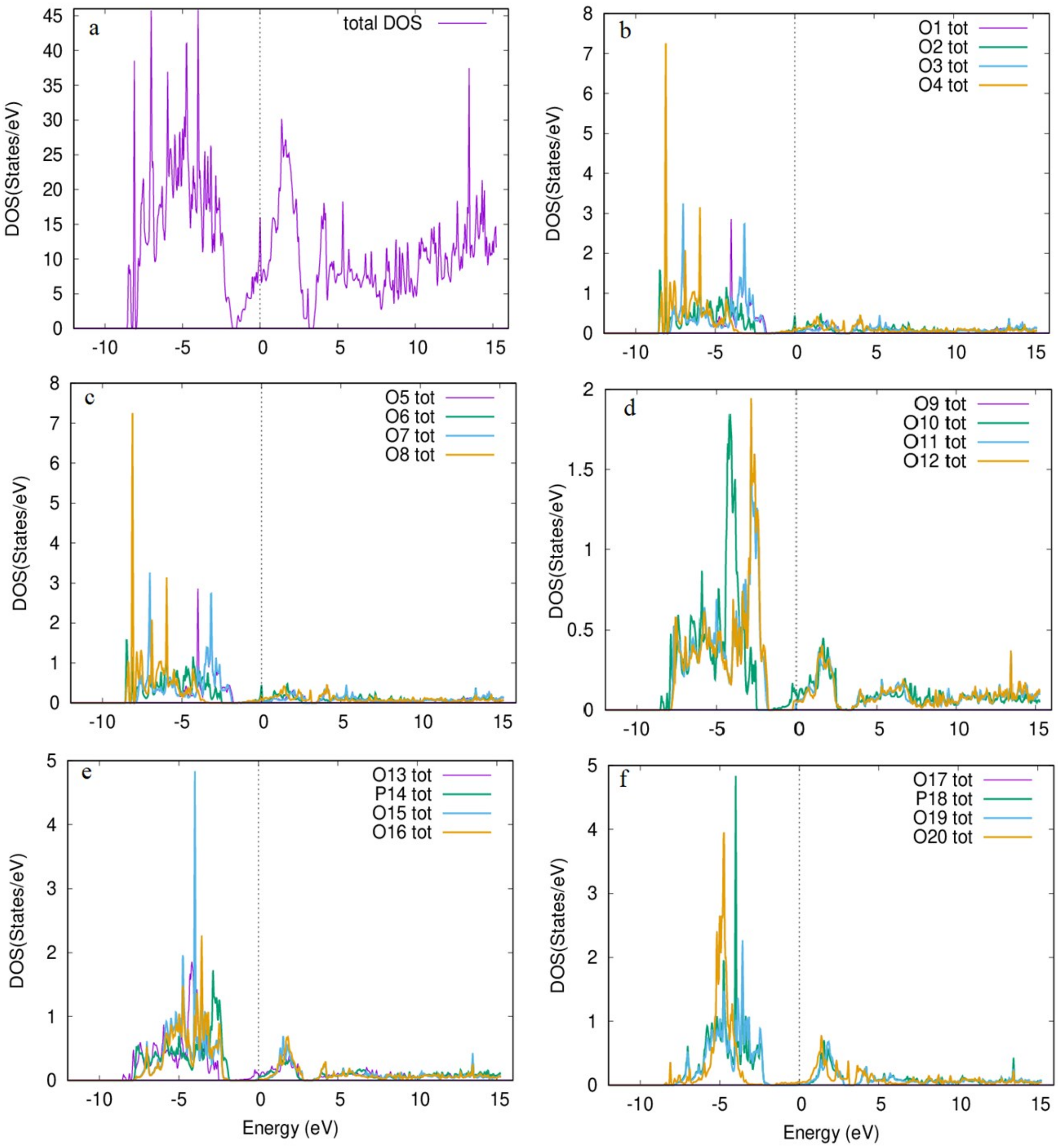

Figure S12 Total Density of states for O in Vacancy 5 structure of molybdenum trioxide (MoO_3) supercell
